# The *Neurospora crassa dfg5* and *dcw1* Genes Encode α-1,6-Mannanases That Function in the Incorporation of Glycoproteins into the Cell Wall

**DOI:** 10.1371/journal.pone.0038872

**Published:** 2012-06-11

**Authors:** Abhiram Maddi, Ci Fu, Stephen J. Free

**Affiliations:** 1 Department of Periodontics and Endodontics, School of Dental Medicine, State University of New York, University at Buffalo, Buffalo, New York, United States of America; 2 Department of Biological Sciences, State University of New York, University at Buffalo, Buffalo, New York, United States of America; Universidad de Sevilla, Spain

## Abstract

The covalent cross-linking of cell wall proteins into the cell wall glucan/chitin matrix is an important step in the biogenesis of the fungal cell wall. We demonstrate that the *Neurospora crassa* DFG5 (NCU03770) and DCW1 (NCU08127) enzymes function *in vivo* to cross-link glycoproteins into the cell wall. Mutants lacking DFG5 or DCW1 release slightly elevated levels of cell wall proteins into their growth medium. Mutants lacking both DFG5 and DCW1 have substantially reduced levels of cell wall proteins in their cell walls and release large amounts of known cell wall proteins into the medium. DFG5 and DCW1 are members of the GH76 family of glycosyl hydrolases, which have specificity to recognize and cleave α-1,6-mannans. A model for incorporation of glycoproteins into the cell wall through the α-1,6-mannan core of the N-linked galactomannan is presented. In this model, DFG5 and DCW1 recognize the N-linked galactomannan present on glycoproteins and cross-link it into the cell wall glucan/chitin matrix.

## Introduction

The cell wall is critical to the survival and growth of fungal cells. It is a dynamic structure that changes in response to environmental conditions and developmental processes.

It is created by the cross-linking of glucans, chitin, and cell wall proteins together into a three-dimensional network. The glucans and chitin are initially synthesized as linear polymers and extruded into the cell wall space during their synthesis. Plasma membrane-associated glucan synthase complexes and chitin synthases utilize intracellular UDP-glucose and UDP-N-acetylglucosamine respectively as substrates to add sugars to the reducing end of the growing polysaccharides. In addition to chitin (a polymer of β-1,4-N-acetylglucosamines), a number of different glucan types have been found in fungal cell walls, including β-1,3-glucose polymers, β-1,6-glucose polymers, polymers having a mixture of β-1,3/β-1,4-glucose linkages, and α-1,3-glucose polymers [1], [2], [3], [4]. Mutants of chitin synthases, β-1,3-glucan synthases, β-1,6-glucan synthases, and α-1,3-glucan synthases demonstrate the importance of the polymers for cell wall biogenesis [4], [5]. As these polysaccharides are extruded into the cell wall space, they are cross-linked together by a group of enzymes having glucanase, chitinase, and glycosyl transferase activities [6], [7], [8]. These cross-linking enzymes are encoded by multi-gene families, which provide the fungi with a number of cell wall cross-linking enzymes having overlapping specificities and a “built-in redundancy” that help insure that the cell wall polymers are effectively cross-linked together.

The *Neurospora crassa* cell wall contains a characteristic array of glycoproteins [9], [10]. These integral cell wall glycoproteins include cross-linking enzymes needed for cell wall biogenesis, sensors for signal transduction pathways, cell wall “structural proteins”, and proteins that provide cell walls with cell type-specific characteristics [2], [3]. These cell wall proteins are cross-linked into the cell wall matrix. Many, but not all, of these proteins are produced as GPI-anchored proteins (glycosylphosphostidylinositol-anchored proteins) [10]. The cell wall proteins have signal peptides and are produced by ER-associated ribosomes. They follow the canonical secretory pathway through the Golgi apparatus and are released into the cell wall space by exocytosis. Previous studies in *Saccharomyces cerevisiae* and *Candida albicans* have shown that β-1,6-glucans can be used to cross-link the oligosaccharides associated with the GPI-anchor into the chitin/glucan matrix, which effectively incorporates GPI-anchored proteins into the cell wall [11], [12]. However, *N. crassa* and *Aspergillus fumigatus* lack β-1,6-glucans, so these fungi must use a different mechanism to covalently cross-link proteins into the cell wall matrix. Recently, we demonstrated that the galactomannan oligosaccharide that is post-translationally added to the N-linked oligosaccharides is required for the covalent incorporation of cell wall proteins into the cell wall of *N. crassa* [13]. Mutants affected in galactomannan synthesis release their cell wall proteins into the growth medium, and have major morphological and growth defects as a result of producing a glycoprotein-deficient cell wall. The galactomannan structure consists of an α-1,6-mannan with short side chains containing α-1,2-linked mannoses and β-linked terminal galactofuranose residues [14]. We now report on two newly identified GPI-anchored cross-linking enzymes that function to generate cross-linking between N-linked oligosaccharide-associated galactomannan and the glucan/chitin matrix of the cell wall, and thereby effectively covalently cross-link glycoproteins into the cell wall matrix. These enzymes belong to the GH-76 class of glycosyl hydrolases and glycosyl transferases, which have specificity for α-1,6-linked mannans.

## Materials and Methods

### Strains and Culturing Conditions

*N. crassa* wild type and mutant isolates were maintained on Vogel’s minimal medium with 2% sucrose at room temperature [15]. The *dfg5*, *dcw1*, and *och-1* deletion mutants were generated as part of the Neurospora genome project [16], and obtained from the Fungal Genetics Stock Center (Kansas City, MO). These deletion strains were created by replacing the coding regions of the genes with a hygromycin resistance cassette [16]. Cloning, sequencing, transformation, and complementation experiments were done as described by Colot et al. [16] and Maniatis et al. [17]. Double mutant strains were created by mating the single mutant isolates. The *Δdfg5, his-3* and *Δdcw1, his-3* isolates used for transformation experiments were generated by mating a *his-3* isolate with the deletion mutants. The genetic analyses, including co-segregation analysis, were carried out as described by Davis and DeSerres [15].

### Isolation and Characterization of GH76 Deletion Mutants

As listed in the *N. crassa* e-compendium (www.bioinf.leeds.ac.uk/~gen6ar/newgenelist/genes), the *N. crassa* genome contains 9 genes encoding GH76 family members (GH76-1/NCU02032, GH76-2/NCU04262, GH76-3/NCU08127, GH76-4/NCU6319, GH76-5/NCU09937, GH76-6/NCU02216, GH76-7/NCU03770, GH76-8/NCU00086, and GH76-9/NCU07005). The Fungal Genetics Stock Center maintains a vast collection of *N. crassa* mutants [18]. Deletion mutants for all nine of these were obtained from the Fungal Genetics Stock Center (Kansas City, MO) (NCU02032/FGSC#21230, NCU04264/FGSC#20538 & 20539, NCU08127/FGSC#19642, NCU06319/FGSC#20003, NCU09937/FGSC#18877 & 18878, NCU02216/FGSC#16188, NCU03770/FGSC#21268, NCU00086/FGSC#15969 & 15970, and NCU07005/FGSC#21045 & 21046). The presence of the deletion mutation was verified for each of these mutants by doing two PCR reactions. One reaction utilized a primer set that gave a product from the deletion mutant genome, in which the gene of interest had been replaced by a hygromycin-resistance cassette. The second PCR reaction gave a product from the wild type copy of the gene, but not from the genome of a deletion mutant. These primers are listed in Table S1. Mutants for all nine genes were also subjected to a morphology and cell wall stress analysis. The morphology of these strains was assessed by growing them in Petri dishes on Vogel’s sucrose medium and examining the gross colony morphology and the morphology of the leading hyphae edge. A more careful examination for cellular morphological differences was carried out by growing the isolates between two sheets of cellophane and examining the hyphae with a differential interference contrast (DIC) microscope [19].

### Cell Wall Stress Tests

To determine if the deletion mutants were affected in the integrity of the cell wall, cell wall stress tests were carried out as previously described [13]. The deletion mutants were grown on 3 ml slants of Vogel’s sucrose agar medium or in Vogel’s liquid medium containing the stress reagents. The growth of the mutants in the presence of the stress reagents was observed over a 72 hour period of time and compared with the growth of the wild type. The cell wall stress reagents used included 10% NaCl (salt stress), 0.01% sodium dodecyl sulfate (detergent), and 10 µg/ml caspofungin (glucan synthesis inhibitor). The caspofungin was obtained as a gift from Merck Research Laboratories (Rahway, NJ). The isolates were also grown at 18°C and 37°C to test for temperature-sensitive growth phenotypes.

### Coomassie Dye Binding Assay

A Coomassie Brilliant Blue G250 dye binding assay was used to assess the amounts of protein contained in purified cell wall preparations [13]. The assay consists of incubating increasing amounts of cell wall material with a 1× concentration of the dye as specified by the manufacturer (BioRad, Hercules, CA) and allowing the dye to bind to the wall during a 30 min. incubation with gentle agitation at room temperature. Following dye absorption, the cell walls were removed with a centrifugation step and the amount of unbound dye was spectrophotometrically determined as OD_465_. The dye binding assay was performed with duplicate samples.

### Carbohydrate Composition and Linkage Analyses

Carbohydrate composition and linkage analyses were performed at the Complex Carbohydrate Research Center (Univ. of Georgia, Athens, GA). Cell wall samples were permethylated, depolymerized, reduced, and acetylated, and the partially methylated alditol acetates (PMAAs) were analyzed by GC/MS.

### Analysis of Secreted Protein by Nano-LC/MS/MS

The preparation and analysis of secreted proteins were performed as previously described [13]. Samples of secreted protein were digested with trypsin and subjected to nano-LC/MS/MS analysis (Midwest BioServices, Overland Park, KS). The secreted proteins were identified by matching peptide sequences obtained from an MS/MS analysis with the protein sequences from the *N. crassa* genome available at the Broad Institute (www.broad.mit.edu) using the TurboSEQUEST software. Only those proteins with multiple peptides and/or single peptides with an XC (correlation coefficient) value of >2.5 for 2 ions or >3.0 for 3 ions were accepted as accurate identifications in the analysis.

### Protein Secretion Assays

To determine if the mutants were affected in the secretion of cell wall proteins, wild type and mutant cells were grown in 250 ml of Vogel’s liquid medium with 2% sucrose for 18 to 20 hours in a shaking incubator at room temperature. The cells were harvested by filtration over a Buchner funnel and the medium collected. Trichloroacetic acid (TCA) precipitation was used to collect the secreted proteins from the medium. Acetone and TCA were added to the medium to a final concentration of 50% acetone and 12.5% TCA, and the proteins were allowed to precipitate for at least 24 hours at −20°C. The secreted proteins were collected by centrifugation, washed twice with −20°C acetone and resuspended in 0.5 ml PBS containing 1% SDS. The samples were placed in a boiling water bath for 10 minutes and the amount of protein present was assayed using the BioRad DC protein assay kit (Hercules, CA). Cytosolic protein was extracted into PBS from mycelia that had been frozen in liquid nitrogen and ground to a fine powder in a mortar and pestle. The concentrations of cytosolic protein were determined with the BioRad protein assay kit. The total amount of secreted protein was divided by the total amount of cytosolic and secreted protein to determine what percentage of the total protein was secreted. Samples from the secreted and the cytosolic protein were used in Western blot experiments.

Western blot experiments were carried out to examine the secretion of two well-known *N. crassa* cell wall proteins, ACW-1 and GEL-1. ACW-1 and GEL-1 are homologs of the *S. cerevisiae* Ecm33p and Gas1p, respectively [10]. These Western blot experiments were carried out as previously described [13]. Polyclonal rabbit antibody directed against amino acids #186–208 of ACW-1 was used at a 1∶5,000 dilution for the detection of ACW-1. Polyclonal rabbit antibody directed against purified GEL-1 was used at a 1∶50,000 dilution for GEL-1 detection. Immunoreactive bands were visualized using a goat anti-rabbit alkaline phosphatase-conjugated secondary antibody (Sigma Aldrich, St. Louis, MO) at a 1∶15,000 dilution.

### Cell Wall Preparations

Cell wall samples were prepared as previously described [13]. The cells were harvested on the Buchner funnel, frozen in liquid N_2_, and ground to a fine powder with a mortar and pestle. PBS buffer was added and a crude cell wall fraction was obtained by a 4,000×G centrifugation step. The cell wall fraction was washed three times with ice-cold PBS buffer and resuspended in 10 ml PBS with 1% SDS. The test tube with the sample was placed in a boiling water bath for 10 minutes to release SDS-soluble cell wall proteins. The cell wall samples were then washed three times with PBS and three times with distilled water to remove SDS from the sample.

### Cloning, Complementation, and RIP Experiments

Cloning experiments were carried out using the pBM61 vector system, which allows for the targeted insertion of cloned genes into the intragenic region downstream of the *his-3* locus [20]. Regions from 1,500 bp upstream of the coding regions to 500 bp downstream of the coding regions for *dfg5* (NCU03770) and *dcw1* (NCU08127) were amplified from wild type genomic DNA and cloned into the pBM61. Primers containing *SpeI* and *EcoRI* sites to facilitate cloning into the vector were used to amplify the wild type *dfg5* gene. Primers containing *NotI* and *BamHI* sites were used to amplify the *dcw1* gene. These primer sequences are given in Table S1. The amplified DNAs and the pBM61 vector were digested with restriction enzymes and ligated together to generate plasmids pBMDFG5 and pBMDCW1, which contained wild type copies of *dfg5* and *dcw1* respectively. These vectors were sequenced to make sure that they contained wild type copies of the genes and then used to transform *Δdfg5*, *his-3* and *Δdcw1*, *his-3* mutants in complementation experiments.

Complementation experiments for *dfg5* were carried out by transforming a *Δdfg5*, *his-3* isolate with the pBMDFG5 vector using the protocol described by Margolin et al. [20], and demonstrating that the wild type copy of the gene restored a wild type colony phenotype to the transformant. Similarly, complementation experiments for *dcw1* were carried out using pBMDCW1 to transform a *Δdcw1, his-3* isolate and showing that the wild type copy of *dcw1* restored wild type hyphal morphology.

As a second way to verify that the *Δdcw1* phenotype was due to loss of *dcw1*, we carried out RIP (repeat induced point mutation) experiments. RIP is a phenomenon in which any DNA sequence that is found in duplicate copies in the *N. crassa* haploid genome is mutated during mating [21]. Multiple C to T and G to A mutations are generated in both copies of the duplicated DNA during RIP. RIP experiments for *DCW1* were done by transforming a *Δdfg5, his-3* mutant with pBMDCW1 to generate a transformant strain having duplicate copies of *dcw1* in a *Δdfg5* background. The transformant was then mated with a *his-3* isolate of the opposite mating type to activate the RIP process. Individual progeny from the mating where screened for the easily assessed *Δdfg5, Δdcw1* double mutant morphology and several such progeny were obtained. PCR amplification and DNA sequence analysis for the *dcw1* gene from a progeny having the double mutant morphology was carried out to verify that the *dcw1* gene had been mutated.

## Results

### Morphological Characterization of the *Δdfg5* and *Δdcw1* Mutants

**Figure 1:**
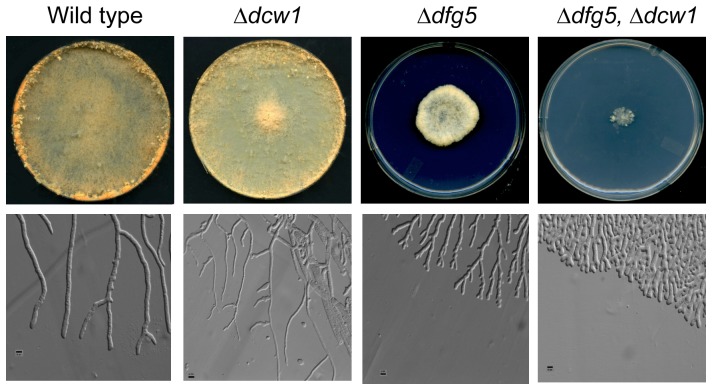
Morphology of *Δdcw1*, *Δdfg5*, and *Δdfg5, Δdcw1* mutants. Colonies of wild type, *Δdcw1*, *Δdfg5*, and *Δdfg5, Δdcw1* mutants growing for 48 hours on Vogel’s sucrose medium in Petri dishes are shown in the upper panels. The lower panels show the morphology of hyphae growing at the edge of the colony growing between sheets of cellophane on a Vogel’s sucrose agar medium. The small black bar in the lower panel pictures is 5 µm in length.

Previous characterization of the *N. crassa Δoch-1* mutant demonstrated the importance of N-linked galactomannan containing an α-1,6-mannose core structure as being necessary for the incorporation of cell wall proteins into the cell wall [13], suggesting that α-1,6-mannanases found in the cell wall space might play a role in cross-linking protein to the glucan/chitin matrix. The *N. crassa* genome contains nine genes in the *gh76* (α-1,6-mannanase) gene family and the library contains deletion mutants for all nine *gh76* genes. We used the PCR primers shown in Table S1 to determine if the targeted *gh76* genes had been deleted in these deletion mutants, and verified that in all cases the strains had the targeted deletions. To determine whether any of the α-1,6-mannanase genes might be required for the incorporation of cell wall proteins into the cell wall glucan/chitin matrix, deletion mutants for all nine genes were tested for morphological phenotypes, for cell wall stability phenotypes, and for the release of cell wall proteins into the growth medium. Two of the mutants, Δ*gh76-7* (NCU03770) and Δ*gh76-3* (NCU08127) had altered morphology. These genes are homologs of the previously identified *S. cerevisiae* DFG5 and DCW1 genes, and we have opted to designate NCU03770 as *dfg5* and NCU08127 as *dcw1* to reflect their relationship to the yeast genes. *Δdfg5* (NCU03770) had a spreading colonial phenotype that was easily recognized visually (Figure 1). *Δdfg5* could also be easily distinguished from the wild type on the basis of its cellular morphology as assessed under the microscope. The mutant hyphae were irregular in diameter and had a dichotomous branching pattern, which was quite different from the subapical branching pattern characteristic of the wild type (Figure 1). *Δdcw1* had a more subtle phenotype. It had a normal gross morphology, but microscopic examination of the hyphal morphology showed that the hyphae produced at the edge of a growing colony were thinner in diameter than the wild type hyphae (Figure 1). Co-segregation experiments demonstrated that the *dfg5* gross morphology phenotype and the *dcw1* hyphal phenotype both co-segregated with the deletion mutations, suggesting that the deletion mutations were responsible for the mutant phenotypes.

A series of double mutants were prepared by mating *Δdfg5* with each of the other *gh76* deletion mutants. We found that the *Δdfg5, Δdcw1* double mutant had a significantly more severe phenotype than either of its parents (Figure 1), while the other double mutants were indistinguishable from the *Δdfg5* parent. The double mutant grew in a much more restricted colonial manner than the *Δdfg5* parent, and microscopic examination of the hyphal morphology showed that the double mutant had a more tightly restricted dichotomous branching pattern (Figure 1).

The susceptibility of each of the *gh76* mutants as well as the *Δdfg5, Δdcw1* double mutant to cell wall perturbation reagents was tested to determine if the mutants had weakened cell walls (Table S2). The ability of the mutants to grow at the low and high temperatures for *N. crassa* growth was also tested. None of the single gene deletion mutants showed susceptibility to the tested cell wall perturbation reagents, but the *Δdfg5* grew very slowly at 18°C and would be classified as having a temperature-sensitive growth phenotype. However, the *Δdfg5, Δdcw1* double mutant showed clear evidence of having a weakened cell wall. The double mutant was susceptible to the presence of low concentrations of SDS and caspofungin. It was also unable to grow at 18°C. We concluded that the double mutant has a weakened cell wall and that the *dfg5* and *dcw1* genes encode α-1,6-mannanases that are required for the synthesis of a normal cell wall.

### Complementation and RIP Experiments Demonstrate *Δdfg5* and *Δdcw1* are Responsible for the Mutant Phenotypes

**Figure 2:**
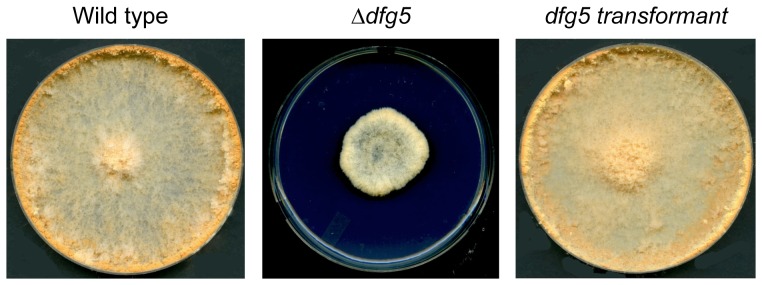
The wild type copy of *dfg5* complements the *Δdfg5* mutation. Colonies of wild type, *Δdfg5*, and a *Δdfg5* mutant that has been transformed with a wild type copy of the *dfg5* gene (labeled as transformant) are shown. The colonies were inoculated in the middle of Petri dishes containing Vogel’s sucrose medium and grown for 48 hours at room temperature.

To demonstrate that deletions of *dfg5* and *dcw1* are responsible for creating the mutant phenotypes, we carried out transformation experiments in which wild type copies of the genes were used to complement the deletion mutations. As described in the Materials and Methods, the wild type copies of the genes were targeted for insertion into the intragenic region downstream of the *his-3* gene. We found that the insertion of a wild type copy of *dfg5* into the genome of the *Δdfg5* mutant complemented the morphological phenotype (Figure 2). We also found that the insertion of a wild type copy of *dcw1* into the *Δdcw1* genome complemented the hyphal morphology phenotype. We conclude that the phenotypes were caused by the deletions and that DFG5 and DCW1 are required for the formation of a normal cell wall.

**Figure 3:**
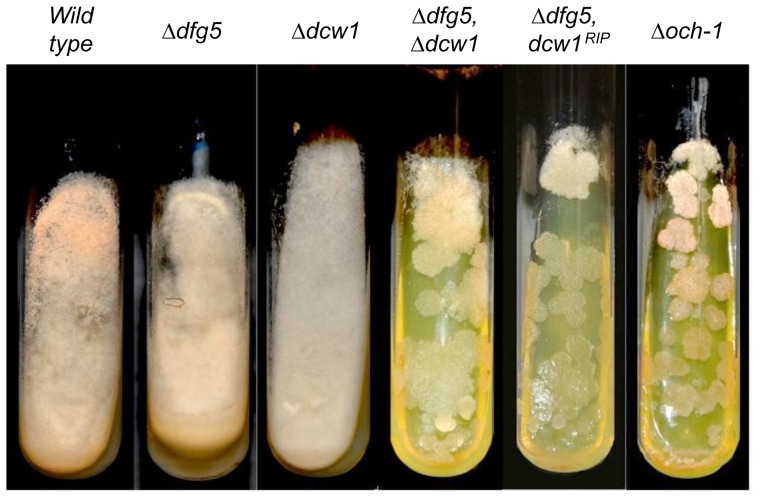
RIP mutation of *dcw1* in the *Δdfg5* background recreates the *Δdfg5, Δdcw1* phenotype. Slants containing Vogel’s sucrose medium were inoculated with mutant and wild type isolates and grown for 48 hours at room temperature. The isolates shown include: 1) wild type (WT), 2) *Δdfg5* mutant, 3) *Δdcw1* mutant, 4) *Δdfg5, Δdcw1* double mutant, 5) *Δdfg5, dcw1RIP* mutant, 6) *Δoch-1* mutant.

In addition to doing the complementation experiment, we also created RIP mutants of *dcw1* to demonstrate that mutation of *dcw1* gives rise to the *dcw1* mutant phenotype we observed. These RIP experiments were done in a *Δdfg5* background because the *Δdfg5, Δdcw1* double mutant has an easily recognized colonial morphology. RIP is a phenomenon in which multiple mutations can be generated in cloned genes [21]. As shown in Figure 3, a *Δdfg5, dcw1^RIP^* double mutant is indistinguishable from the *Δdfg5, Δdcw1* double mutant. One of these RIP alleles, *dcw1^RIP2^*, was PCR amplified and sequenced. It was found to have 173 mutations, including 11 stop codons, and mutations altering the branch sites in two introns within the gene (Genbank accession # JQ520137). The first stop codon was at amino acid 30, well before the predicted catalytic region of the 478 amino acid DCW1. These results verify that mutations in *dcw1* are responsible for the mutant phenotypes. The morphological characteristics of the *Δdfg5, Δdcw1* and the *Δdfg5, dcw1^RIP^* double mutants closely resemble those found for the *Δoch-1* mutant (Figure 3), which has been shown to be defective in incorporating cell wall proteins into the cell wall [13].

### The *Δdfg5, Δdcw1* Double Mutant is Defective in Incorporating Cell Wall Proteins into the Wall

**Figure 4:**
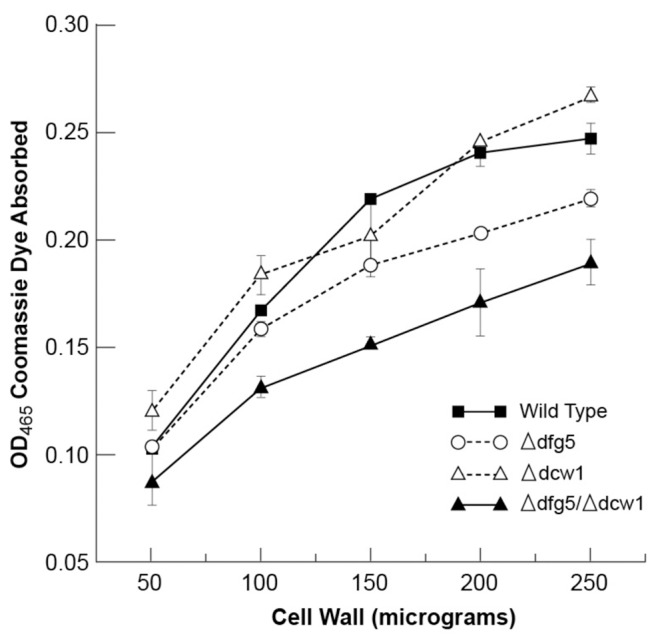
The *Δdfg5, Δdcw1* cell wall is deficient in cell wall protein. Coomassie brilliant blue dye assay of cell wall protein. Increasing amounts of wild type, *Δdfg5*, *Δdcw1*, and *Δdfg5, Δdcw1* cell walls were incubated in a solution of Coomassie Blue, and the amounts of dye absorbed by the cell walls were determined.

To determine if the *Δdfg5, Δdcw1* double mutant was impaired in the incorporation of protein into the cell wall, we used the Coomassie Blue binding assay described in Materials and Methods to examine the protein levels in purified cell walls from the wild type, *Δdfg5*, *Δdcw1*, and double mutant isolates (Figure 4). Although the assay isn’t linear with the amount of cell wall used, Figure 4 clearly shows that the *Δdfg5, Δdcw1* cell wall absorbs much less dye than the wild type cell wall and that *Δdfg5* may also have a reduced amount of cell wall protein. The levels of cell wall protein in the *Δdcw1* cell wall appear to be comparable to those in the wild type cell wall. We conclude that the *Δdfg5, Δdcw1* cell wall has less protein in it than the wild type cell wall.

To further demonstrate that the *Δdfg5*, *Δdcw1*, and *Δdfg5, Δdcw1* mutants were affected in the ability to incorporate proteins into their cell walls, the release of proteins into the medium was assessed as described in the Materials and Methods. We found the *Δdfg5* and *Δdcw1* mutants released 1.6 and 1.8 times as much protein into the medium as the wild type. The double mutant released 17.7 times as much protein as the wild type (The wild type cells released 0.1% of their total protein into the medium while the double mutant released 1.77% of its total protein into the medium). We conclude that both mutants are affected in the ability to retain protein and that the double mutant secretes a significantly larger amount of protein than the wild type cell. These results from the *Δdfg5, Δdcw1* double mutant closely mirror the results from the *Δoch-1* mutant [13].

**Figure 5:**
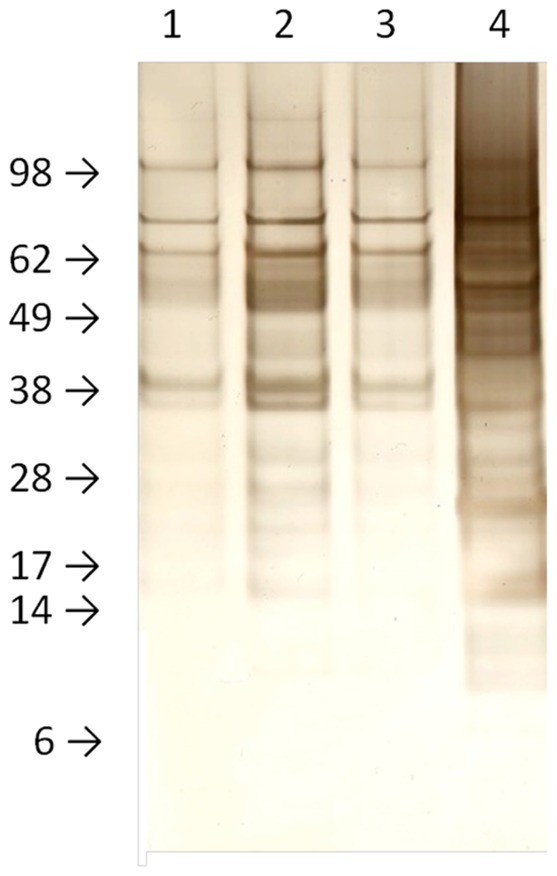
SDS PAGE analysis of secreted protein shows that *Δdfg5, Δdcw1* secretes large amounts of protein. Samples of secreted protein representing the amount of protein secreted from cells containing 300 µg of cytosolic protein were subjected to SDS PAGE and stained with silver stain. Lane 1, proteins secreted by wild type cells. Lane 2, proteins secreted by *Δdcw1*. Lane 3, proteins secreted by *Δdfg5*. Lane 4, proteins secreted by *Δdfg5, Δdcw1*.

To examine which proteins are being released by the wild type and mutant cells, an SDS PAGE analysis was carried out. Figure 5 shows a silver stained gel containing the secreted protein samples from wild type and mutant cells. Each lane in the gel contains the amount of secreted protein released by cells having 300 µg worth of cytosolic protein. As is clear from the gel, the *Δdfg5, Δdcw1* double mutant releases large amounts of protein into the medium. An examination of the proteins in the gel suggests that the wild type and single deletion mutants released largely the same array of proteins, while the double mutant may be releasing a few additional proteins into the medium.

A proteomic analysis was carried out to identify some of proteins secreted by the wild type and *Δdfg5, Δdcw1* cells. Samples of secreted proteins were digested with trypsin and the released peptides analyzed by nano-LC/MS/MS (Midwest BioServices, Overland Park, KS). A larger number of proteins were identified in the *Δdfg5, Δdcw1* sample than in the wild type sample, and we often identified a larger number peptides for these proteins in the *Δdfg5, Δdcw1* sample (Table S3). These findings are consistent with the release of large amounts of cell wall protein into the growth medium by *Δdfg5, Δdcw1*.

### The *Δdfg5, Δdcw1* Double Mutant Releases known Cell Wall Proteins to the Medium

**Figure 6:**
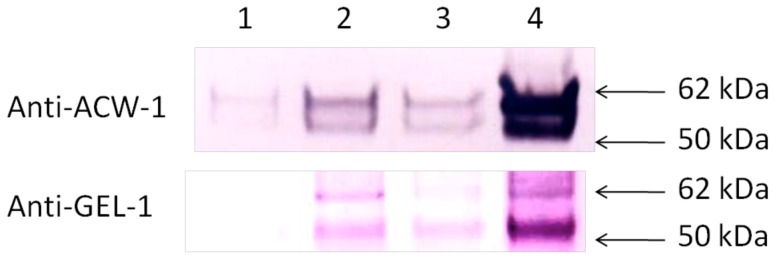
Western blot analysis of ACW-1 and GEL-1 secretion in wild type and mutant cells. Western blot analyses using antibodies directed against ACW-1 (top panel) and GEL-1 (bottom panel) were carried out on the secreted protein from cells having 300 µg of cytosolic protein. ACW-1 and GEL-1 are two well-characterized *N. crassa* cell wall proteins. Lane 1, wild type secreted protein. Lane 2, *Δdcw1* secreted protein. Lane 3, *Δdfg5* secreted protein. Lane 4, *Δdfg5, Δdcw1* secreted protein.

To look at the release of known cell wall proteins into the medium, antibodies directed against two well-known cell wall proteins, ACW-1 and GEL-1, were used in Western blot experiments. The amounts of these two proteins in the medium were examined as described in Materials and Methods. As is evident in Figure 6, both single gene deletion mutants secrete slightly elevated levels of ACW-1. However, the double mutant clearly released high levels of ACW-1 into the medium. The higher molecular weight band in Figure 6 is the same size as the ACW-1 *in transit* to the cell wall (Figure S1), and the lower molecular weight band is thought to be generated by proteolysis of the secreted ACW-1. Analysis of the levels of intracellular ACW-1 showed that intracellular levels of ACW-1 were comparable in all four strains (Figure S1). The increased level of secreted ACW-1 in the double mutant cannot be ascribed to an increased rate of ACW-1 synthesis by the mutant, but must come from a difference in the ability of the mutant to covalently incorporate ACW-1 into the cell wall matrix. The fact that the levels of released ACW-1 in the double mutant is much more than the addition of the amounts released by the single mutants suggests that DFG5 and DCW1 are each capable of cross-linking ACW-1 into the wall, and when both enzymes are lost the incorporation of ACW-1 into the cell wall is severely compromised. The data suggests that there is redundancy in the functions of DFG5 and DCW1. Having a group of glucanases with redundant activities cross-linking the glucan/chitin cell wall matrix is a common theme in fungal cell wall biogenesis [2], [3], [5]. Figure 6 also shows a similar experiment in which the levels of released GEL-1, another well-known *N. crassa* cell wall protein [10] were examined by Western blot analysis. As was the case for ACW-1, *Δdfg5, Δdcw1* released large amounts of GEL-1 into the medium. We conclude that the DFG5 and DCW1 encoded mannanases which are required for the efficient cross-linking of cell wall proteins into the cell wall.

### Cell Wall Sugar Analysis Further Substantiates that *Δdfg5, Δdcw1* has a Reduced Level of Glycoprotein

Analyses of the *N. crassa* cell wall carbohydrate has shown that the wall contains large amounts of 1,3-linked and 1,4-linked glucose, with lesser amounts of mannose and galactose residues [13]. The glucose is derived from the glucans within the wall, while the mannose and galactose have been shown to be, in large part, derived from the post-translational modifications found on glycoproteins [13]. In Δ*och-1*, which is unable to incorporate glycoprotein into the cell wall, the levels of galactose and mannose residues are dramatically reduced from those found in the wild type cell wall [13]. We carried out a similar analysis of the sugars and their linkages in the *Δdfg5, Δdcw1* double mutant and found that the levels of mannose and galactose were dramatically reduced from those found in the wild type cell wall (Table 1). The results from the cell wall analysis for *Δdfg5, Δdcw1* are similar to those previously found in the *Δoch-1* cell wall. The reduced levels of cell wall mannose and galactose further substantiate the conclusion that DFG5 and DCW1 function in cross-linking cell wall proteins into the cell wall matrix.

**Table 1:** Linkage analysis of the carbohydrates present in wild type and mutant cell walls.

Glycosyl Residue	Wild type cell wall (% of totalsugar in analysis)	*Δdfg5, Δdcw1* cell wall (% of totalsugar in analysis)
Terminally linked mannopyranosyl (t-Man)	3.4%	0.7%
2-linked mannopyranosyl (2-Man)	5.4%	1.1%
6-linked mannopyranosyl (6-Man)	2.0%	0.5%
2,3-linked mannopyranosyl (2,3-Man)	1.7%	0.0%
2,6-linked mannopyranosyl (2,6-Man)	2.3%	0.0%
**Total Mannose residues**	**14.8%**	**2.3%**
Terminally linked galactofuranosyl (t-Galf)	6.8%	1.1%
Terminally linked galactopyranosyl (t-Gal)	1.6%	1.1%
4-linked galactopyranosyl (4-Gal)	2.5%	1.3%
2,3-linked galactopyranosyl (2,3-Gal)	0.0%	0.3%
6-linked galactopyranosyl (6-Gal)	0.8%	0.0%
**Total Galactose residues**	**11.7%**	**3.8%**
Terminally linked glucopyranosyl (t-Glc)	9.1%	6.2%
3-linked glucopyranosyl (3-Glc)	40.1%	41.2%
4-linked glucopyranosyl (4-Glc)	18.9%	42.7%
3,4-linked glucopyranosyl (3,4-Glc)	1.3%	0.5%
2,3 linked glucopyranosyl (2,3-Glc)	1.8%	1.5%
3,6 linked glucopyranosyl (3,6-Glc)	0.9%	0.5%
4,6 linked glucopyranosyl (4,6-Glc)	0.7%	1.3%
**Total Glucose residues**	**72.8%**	**92.6%**

The amounts of the different sugar linkages found in the analysis are given as a percentile of the total carbohydrate. The total percentage of mannose, galactose and glucose in the analysis was determined by the addition of all of the different linked mannose, galactose and glucose residues.

## Discussion

The cell wall is a vital and essential structure for filamentous fungi. It protects the cell from environmental stresses and allows the cell to assess environmental changes. Cell wall proteins have been shown to be important determinants of morphology, virulence and adhesion [2], [3], [4], [5]. Cell wall proteins include sensors and receptors for signal transduction pathways, as well as structural proteins, adhesins, and cell wall biosynthetic enzymes. These proteins are produced by ER-associated ribosomes and pass through the canonical secretory pathway. Once secreted into the cell wall space, many cell wall proteins become covalently cross-linked into the cell wall chitin/glucan matrix.

We report on the characterization of two *N. crassa* cell wall proteins, DFG5 and DCW1. These α-1,6-mannanases are homologs of the *S. cerevisiae* Dfg5p and Dcw1p cell wall proteins. DFG5 and DCW1 have N-terminal signal peptides as well as an ω site for the addition of a GPI-anchor, suggesting that they function in the cell wall space. Our data shows that loss of DFG5 and DCW1 gives rise to a colonial phenotype and sensitivity to cell wall perturbation reagents, indicating that DFG5 and DCW1 function in cell wall biogenesis. We found that total cell wall protein was reduced in a *Δdfg5, Δdcw1* cell. Cell wall sugar linkage analysis showed that the double mutant cell wall had decreased levels of mannose and galactose. Mannose and galactose are found in the post-translational modifications of cell wall glycoproteins, and the decreased levels of these sugars substantiates that the double mutant was defective in the incorporation of protein into the wall. We also showed that *Δdfg5, Δdcw1* released large amounts of protein into the medium, including large amount of ACW-1 and GEL-1, two well-characterized cell wall proteins. All of these findings clearly demonstrate that DFG5 and DCW1 are required for the efficient incorporation of glycoprotein into the *N. crassa* cell wall.

Our data indicates that DFG5 and DCW1 have some redundancy in their functions, such as the ability to cross-link ACW-1, GEL-1, and a number of other proteins into the cell wall (Figure 5 and Figure 6). However, since *Δdfg5* and *Δdcw1* have different phenotypes (Figure 1), there may be some proteins that are preferentially incorporated into the cell wall by DFG5 or DCW1. Loss of these proteins could be responsible for the differences between the *Δdfg5* and *Δdcw1* phenotypes.

Previous work has shown that the *N. crassa Δoch-1* mutant is unable to incorporate cell wall proteins into the cell wall matrix [13]. OCH-1 catalyzes the addition of a mannose residue to N-linked oligosaccharides, and is the first step in the biosynthetic pathway for outer chain mannans in *S. cerevisiae* and for an N-linked galactomannan in *N. crassa* [13], [22], [23], [24]. The galactomannan is thought to contain an α-1,6-mannan core chain with α-1,2-mannose containing side chain that are terminated by galactofuranose residues [14]. In this report, we show that DFG5 and DCW1, two cell wall α-1,6-mannanases, are also required for the incorporation of cell wall protein into the cell wall glucan/chitin matrix. The data from this report, combined with the data from the OCH-1 study, suggests that DFG5 and DCW1 function by recognizing N-linked galactomannans, cleaving the α-1,6-mannan backbone, and cross-linking the N-linked galactomannan into the glucan/chitin matrix. In so doing, they would effectively cross-link the glycoprotein into the cell wall structure. This model for the incorporation of glycoproteins into the cell wall is interesting in that the cross-linking of protein into the wall occurs by essentially the same mechanism used to cross-link the glucan/chitin matrix. Several cell wall glucanases and chitinases have been shown to function in cross-linking the glucan/chitin matrix [2], [3], [5]. The best characterized of these enzymes are the Gas1p/GEL1 enzymes, which have been shown to be able to cleave and join β-1,3-glucans [8]. Many of these enzymes are encoded by multi-gene families, and multiple members of these families are expressed providing the cell with a built-in biosynthetic redundancy. One appealing feature of the mechanism we have uncovered for incorporating glycoproteins into the cell wall though N-linked galactomannans is that it would be relatively easy for fungal cells, expressing a large number of such sugar polymer hydrolase/transferase enzymes, to evolve the ability to cross-link the galactomannan into the cell wall matrix. A small change in substrate specificity for one of the enzymes involved in cross-linking the matrix glucans and chitins would be sufficient for the creation of an enzyme capable of cross-linking protein into the cell wall.

DFG5 and DCW1 encoding genes have been identified in the genomes of a number of fungi, and the encoded proteins are well conserved. Table S4 shows the alignment of DFG5 and DCW1 with the related genes from *S. cerevisiae*, *C. albicans* and *A. fumigatus*. The genes were first identified in *S. cerevisiae*, and named DFG5 (Defective for Filamentous Growth) and DCW1 (Defective Cell Wall) [25], [26]. Mutants lacking these genes have been studied in both *S. cerevisiae* and *C. albicans* [25], [26], [27], [28]. The deletion of both genes is lethal in these organisms [26], [28]. In *C. albicans*, Dfg5p was shown to be localized to the plasma membrane and cell wall [27]. *C. albicans* mutants lacking Dfg5p and Dcw1p were found to be defective in hyphal formation [28]. Because the expression of a hyphal-specific cell wall protein was affected in DFG5 mutants, Spreghini et al. [28] suggested that Dfg5p might function in transmitting an extracellular signal needed for the expression of hyphal-specific genes. The *C. albicans* DFG5 mutant has been shown to be sensitive to caspofungin, suggesting that Dfg5p functions in cell wall biogenesis [29]. Kitagaki et al. [26] demonstrated that the *S. cerevisiae* DFG5 and DCW1 genes were needed for cell wall biogenesis and that Cwp1p, a GPI-anchored cell wall protein, was secreted in the absence of Dfg5p and Dcw1p. Gonzalez et al. [30] also demonstrated that mutants lacking Dfg5p and Dcw1p secreted GPI-anchored proteins. In *S. cerevisiae*, GPI-anchored proteins have been shown to be cross-linked into the cell wall through β-1,6-glucan polymers [12], and Kitagaki et al. [26] speculated that Dfg5p and Dcws1p were involved in this cross-linking activity. Our hypothesis differs from that presented by Kitagaki et al., in that we propose that the *N. crassa* DFG5 and DCW1 function to cross-link the α-1,6-mannan core of the N-linked galactomannan present on glycoproteins into the cell wall matrix.

Studies in other fungi suggest that the incorporation of protein into the cell wall matrix can occur by multiple mechanisms. In *S. cerevisiae*, Lu et al. [12] found that β-1,6-glucan can function to cross-link the GPI-anchor found on some cell wall proteins into the cell wall glucan. *N. crassa* cell walls do not contain β-1,6-glucan [10], and the genome lacks the genes for its synthesis [16]. Our studies on the *N. crassa Δoch-1* mutant demonstrate that it is unable to incorporate cell wall protein into the cell wall [13], but studies in *Aspergillus fumigatus* indicate that mutation of the och1 gene in that species does not give rise to a mutant phenotype [31]. These findings suggest that the fungi may have evolved multiple ways to cross-link the elements of their cell walls so as to provide for functional redundancy and robustness in cell wall biogenesis. The cell wall is a critical structure for the survival of the fungi, and providing multiple cross-linking mechanisms to insure the formation of a strong cell wall may have been selected for in different fungal lineages. The robustness and redundancy in cell wall biosynthetic functions is highlighted by the presence of 133 encoded glycosyl hydrolases in the *N. crassa* genome, as noted in the *N. crassa* e-compendium. Other filamentous fungi also have large numbers of glycosyl hydrolase genes. Most of these genes encode proteins with signal peptides and many also have GPI-anchor signals at their encoded C termini, suggesting that most of these enzymes are found in the cell wall and function in cell wall biogenesis. Our laboratory has carried out a functional analysis of 45 gene deletion mutants for *N.* *crassa* glycosyl hydrolases. With the exception of *dfg5*, we have found that the deletion of individual glycosyl hydrolases does not give rise to dramatically altered phenotypes (unpublished data).

Our results indicate that DFG5 and DCW1 function in cross-linking proteins into the cell wall, and that they have a redundancy in function. In addition to DFG5 and DCW1, the *N. crassa* genome has another seven related α-1,6-mannanase genes. Some of these might function as “minor” cross-linking enzymes during vegetative growth, and this could explain why the *N. crassa* double mutant is viable while the equivalent double mutants for *S. cerevisiae* and *C. albicans* are lethal. Another possibility is that some of these other α-1,6-mannanases function in different cell types during the Neurospora life cycle. One common theme that emerges from the study of fungal cell walls is that by producing a large number of cell wall cross-linking enzymes with overlapping functions, the fungus insures that it will be able to create a strong cell wall under a wide variety of environmental conditions.

## Supporting Information

Figure S1 **Wild type and mutant cell contain comparable levels of ACW-1** ***in transit*** **to the cell wall.** 30 ugr of cytosolic proteins from wild type (lane 1), *Δdcw1* (lane 2), *Δdfg5* (lane 3), and the *Δdfg5, Δdcw1* double mutant (lane 4) were subjected to a Western blot analysis for ACW-1.
(DOC)

Table S1 Primers used for cloning experiments. Sequences for each of the primers used in cloning experiments are shown.
(DOC)

Table S2 Sensitivity to Stress Conditions. Conidia from wild type, *Δdfg5*, *Δdcw1*, and the *Δdfg5, Δdcw1* double mutant were used to inoculate test tubes containing 3 ml of Vogel’s minimal medium with 2% sucrose. Individual tubes were supplemented with caspofungin, SDS, and NaCl as indicated, or placed in incubators at 18°C and 37°C. The growth of the culture was assessed after 72 hours of incubation.
(DOC)

Table S3 Proteomic analysis of *Neurospora crassa* proteins released into Vogel’s sucrose liquid growth medium by wild type and *Δdfdg5, Δdcw1* vegetative hyphae. All of the peptides identified in a nano-LC/MS/MS analysis of secreted proteins from vegetative wild type cells and *Δdfg5, Δdcw1* cells are listed in the middle column. The proteins from which these peptides were derived were identified using the TurboSEQUEST software to search the proteins encoded by the *N. crassa* genome at the Broad Institute and NCU#s are shown in the first two columns. The location of the peptide within the identified protein is given in the fourth column. Whether the peptides were found in the secreted proteins from wild type cell (WT) or from the *Δdfg5, Δdcw1* (mutant) secreted proteins is denoted in the last two columns. A * by the protein name denotes a putative GPI-anchored protein.
(DOC)

Table S4 Alignments for DFG5 and DCW1. Alignments of DFG5 and DCW1 from *N. crassa*, *S. cerevisiae*, *C. albicans*, and *A. fumigatus* using CLUSTAL W (1.83) multiple sequence alignment.
(DOC)
