# Supplementary material for: The Neurospora crassa dfg5 and dcw1 Genes Encode α-1,6-Mannanases That Function in the Incorporation of Glycoproteins into the Cell Wall
Source: PLoS One. 2012 Jun 11;7(6):e38872. doi: 10.1371/journal.pone.0038872 (PMC3372484; doi:10.1371/journal.pone.0038872)
Supplement: Figure S1 — Wild type and mutant cell contain comparable levels of ACW-1 in transit to the cell wall. 30 ugr of cytosolic proteins from wild type (lane 1), Δdcw1 (lane 2), Δdfg5 (lane 3), and the Δdfg5, Δdcw1 double mutant (lane 4) were subjected to a Western blot analysis for ACW-1. (DOC) [file pone.0038872.s001.doc]

**Supplemental Figure 1**

ACW-1 expression levels in wild type and mutant isolates.


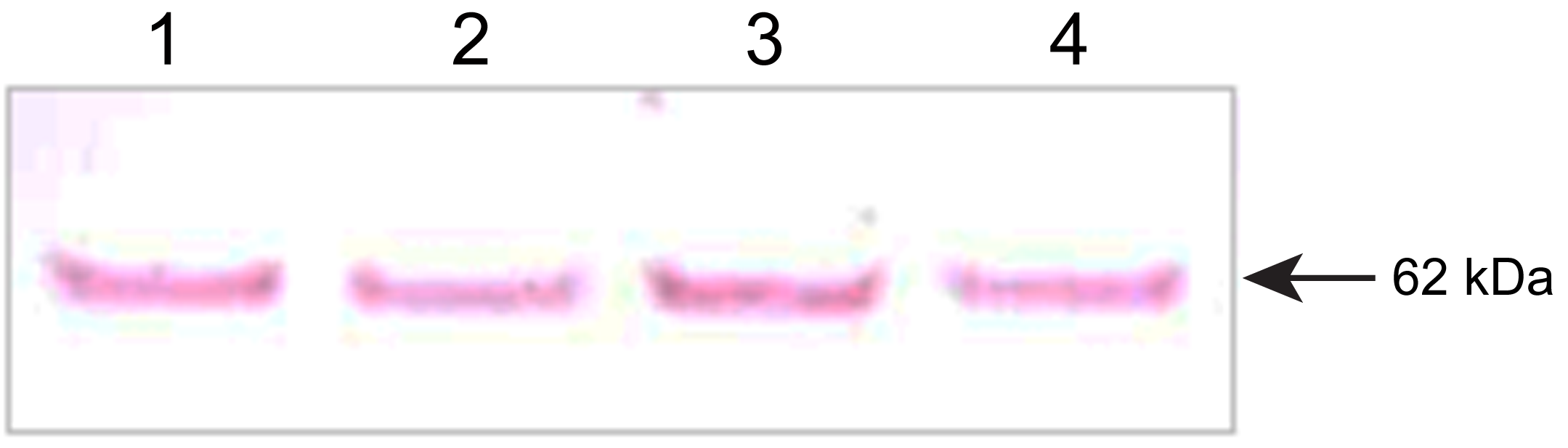


**Supplemental Figure 1.** Wild type and mutant cell contain comparable levels of ACW-1 *in transit* to the cell wall. 30 ugr of cytosolic proteins from wild type (lane 1), *Δdcw1* (lane 2), *Δdfg5* (lane 3), and the *Δdfg5, Δdcw1* double mutant (lane 4) were subjected to a Western blot analysis for ACW-1.
