# Supplementary material for: The Neurospora crassa dfg5 and dcw1 Genes Encode α-1,6-Mannanases That Function in the Incorporation of Glycoproteins into the Cell Wall
Source: PLoS One. 2012 Jun 11;7(6):e38872. doi: 10.1371/journal.pone.0038872 (PMC3372484; doi:10.1371/journal.pone.0038872)
Supplement: Table S1 — Primers used for cloning experiments. Sequences for each of the primers used in cloning experiments are shown. (DOC) [file pone.0038872.s002.doc]

**Table S1**

primers used for cloning experiments. Sequences for each of the primers used in cloning experiments are shown.

| Primer name | Sequence |
| --- | --- |
| DFG5-5’end (SpeI) | ATGCACTAGTCGCATGCAGCATTGCAATCG |
| DFG5-3’end (EcoRI) | CAATGAATTCCAATCTTGTGTGATCAGATC |
| DCW1-5-end (NotI) | ATATGCGGCCGCGCACACTACACTACACTAC |
| DCW1-3’end (BamHI) | CTAGGGATCCGCTTTTGCCATGAGACCC |
| DFG5-coding region forward primer (SpeI) | TTAAACTAGTCGCCATGAGGTGGAATGTTGC |
| glx-1-coding region his6 reverse primer | TGCTTAATGATGGAGGAGATGATGAACCCGATCCGCCATGGTGAT |
| DFG5-coding region his6 forward primer | CGGGTTCATCATCACCACCATCATTAAGCAGCCGGGCGTTGTTGG |
| DFG5-3’UTR reverse primer EcoRI | CAATGAATTCCAATCTTGTGTGATCAGATC |
| Hygromycin reverse primer | CATATGCGCGATTGCTGATCC |
| gh76-1 (NCU02032) forward primer | CCGGCCTACTCTCACTACAATG |
| gh76-1 (NCU02032) reverse primer | CCCCAAAACCCTTGATCATCG |
| gh76-2 (NCU04262) forward primer | CCCATCGATCCATTGTCGTTGC |
| gh76-2 (NCU04262) reverse primer | CCCAGCCACTGTGGTTGATC |
| gh76-3 (NCU08127) forward primer | CCGATCCAACTTAATCGCGTAC |
| gh76-3 (NCU08127) reverse primer | CGCCGGTCAAGTGCCAGTAATC |
| gh76-4 (NCU06319) forward primer | CGTCCGATATTTCCGTCCAC |
| gh76-4 (NCU06319) reverse primer | CCCGTATGGAAGAGCATAGAG |
| gh76-5 (NCU09937) forward primer | CGCCTTGAAGCCAACAATAGTG |
| gh76-5 (NCU09937) reverse primer | CCCACACCTTGTTTGCCCAC |
| gh76-6 (NCU02216) forward primer | CTCCAGCCGTCAAACTGCCC |
| gh76-6 (NCU02216) reverse primer | CAGCTTGCGTGTTGAAGACG |
| gh76-7 (NCU03770) forward primer | CCTTCAAAATACAACCCGTC |
| gh76-7 (NCU03770) reverse primer | CGCCTGCTTGGTGTACAATC |
| gh76-8 (NCU00086) forward primer | CCGCAAGAACTCGGCACACA |
| gh76-8 (NCU00086) reverse primer | CACCAGGCGACATCAGGAGA |
| gh76-9 (NCU07005) forward primer | GCCCAGCTTTGTCCATGACC |
| gh76-9 (NCU07005) reverse primer | CCTGTCCTTGCTCCACCATATG |
