# Supplementary material for: The Neurospora crassa dfg5 and dcw1 Genes Encode α-1,6-Mannanases That Function in the Incorporation of Glycoproteins into the Cell Wall
Source: PLoS One. 2012 Jun 11;7(6):e38872. doi: 10.1371/journal.pone.0038872 (PMC3372484; doi:10.1371/journal.pone.0038872)
Supplement: Table S2 — Sensitivity to Stress Conditions. Conidia from wild type, Δdfg5, Δdcw1, and the Δdfg5, Δdcw1 double mutant were used to inoculate test tubes containing 3 ml of Vogel’s minimal medium with 2% sucrose. Individual tubes were supplemented with caspofungin, SDS, and NaCl as indicated, or placed in incubators at 18°C and 37°C. The growth of the culture was assessed after 72 hours of incubation. (DOC) [file pone.0038872.s003.doc]

**Table S2**

Sensitivity to Stress Conditions

| Strain | Caspofungin | 0.01% SDS | 10% NaCl | 18^o^C | 37^o^C |
| --- | --- | --- | --- | --- | --- |
| Wild type | Growth | Growth | Growth | Growth | Growth |
| *Δdfg5* | Growth | Growth | Growth | **Minimal growth** | Growth |
| *Δdcw1* | Growth | Growth | Growth | Growth | Growth |
| *Δdfg5, Δdcw1* | **No growth** | **No growth** | Growth | **No growth** | Growth |

Testing strains for the ability to grow under different stress conditions. Conidia from wild type, *Δdfg5*, *Δdcw1*, and the *Δdfg5, Δdcw1* double mutant were used to inoculate test tubes containing 3 ml of Vogel’s minimal medium with 2% sucrose. Individual tubes were supplemented with caspofungin, SDS, and NaCl as indicated, or placed in incubators at 18^o^C and 37^o^C. The growth of the culture was assessed after 72 hours of incubation.
