# Supplementary material for: The Neurospora crassa dfg5 and dcw1 Genes Encode α-1,6-Mannanases That Function in the Incorporation of Glycoproteins into the Cell Wall
Source: PLoS One. 2012 Jun 11;7(6):e38872. doi: 10.1371/journal.pone.0038872 (PMC3372484; doi:10.1371/journal.pone.0038872)
Supplement: Table S3 — Proteomic analysis of Neurospora crassa proteins released into Vogel’s sucrose liquid growth medium by wild type and Δdfdg5, Δdcw1 vegetative hyphae. All of the peptides identified in a nano-LC/MS/MS analysis of secreted proteins from vegetative wild type cells and Δdfg5, Δdcw1 cells are listed in the middle column. The proteins from which these peptides were derived were identified using the TurboSEQUEST software to search the proteins encoded by the N. crassa genome at the Broad Institute and NCU#s are shown in the first two columns. The location of the peptide within the identified protein is given in the fourth column. Whether the peptides were found in the secreted proteins from wild type cell (WT) or from the Δdfg5, Δdcw1 (mutant) secreted proteins is denoted in the last two columns. A * by the protein name denotes a putative GPI-anchored protein. (DOC) [file pone.0038872.s004.doc]

**Table S3**

Proteomic analysis of *Neurospora crassa* proteins released into Vogel’s sucrose liquid growth medium by wild type and *Δdfdg5, Δdcw1* vegetative hyphae.

| ***Protein Name/***  ***Description/(Mol.Wt)*** | ***Gene***  ***Locus No.*** | ***Peptide Sequence*** | ***Residues*** | ***WT*** | ***mutant*** |  |
| --- | --- | --- | --- | --- | --- | --- |
| ACW-1/CCG-15* (49 kDa)  ACW-2* (28 kDa)  ACW-3* (28 kDa)  ACW-7* (25 kDa)  ACW-12* (53 kDa)  GH17-3/Glucan-β-glucanase* (49 kDa)  GH16-7/Glycoside hydrolase* (38 kDa)  GH72-5/GEL1* (58 kDa)  GH55-3/GEL2* (85 kDa)  GH28-1/Polygalacturonase precursor (40 kDa)  NCW-1 (74 kDa)  GLA-1/Glucamylase (66 kDa)  ASD-1/SDV-10/ Rhamnogalacturonase  (61 kDa)  Predicted protein (28 kDa)  Predicted protein (21 kDa)  CAT-3/Catalase (80 kDa)  α,β-hydrolase (43 kDa)  GH47-5/α1,2-mannosidase precursor (60 kDa)  GH61-3/Endoglucanase II  (36 kDa)  GH2-4/Glycoside hydrolase  (75 kDa)  GH55-1/Exo-β1,3-glucanase  (81 kDa)  GH55-3/ β1,3-glucanase  (85 kDa)  GH13-1/Alpha-amylase*  (59 kDa)  Βeta-glucosidase (46 kDa)  Tripeptidyl peptidase  (63 kDa)  APR-3/Aspartyl protease 3  (55 kDa)  SPR-7/Serine protease 7  (40 kDa)  PEP-4/Vacuolar protease A  (43 kDa)  Predicted protein (31 kDa)  Predicted protein (31 kDa)  Hypothetical protein  (24 kDa)  Hypothetical protein  (31 kDa)  Hypothetical protein  (15 kDa) | NCU08936  NCU00957  NCU05667  NCU09133  NCU08171  NCU09175  NCU05974  NCU08909  NCU07253  NCU02369  NCU05137  NCU01517  NCU05598  NCU04603  NCU00399  NCU00355  NCU02904  NCU07607  NCU02916  NCU00985  NCU04850  NCU07523  NCU08131  NCU02668  NCU08418  NCU02059  NCU07159  NCU02273  NCU00265  NCU01720  NCU04675  NCU00811  NCU00995 | EVAGDFQLSGPQIITGDLK  IENNPNINSVGSDTLEQIGGEFR  LVETTQLNAVNMLK  LTWQSIGNVPDMGMTQLNK  SMNLDNNQFITK  DATAPITITLMTGPDADHMTPFK  TIASGVTGDSYTWTPEDVPSGTYAFK  FPYVGSAAATTGASSTLSTVTK  KNDVVDPSSGVEVK  DLGAVDLTK  IDVLGGTTPQTLDK  PLTVAAPATTK  LTEQADNVNVTSLSDIFSAAGK  VVSVADVFNDGNHQMTTDSYGR  KLWQHTSDFNDENTTK  HALLVYPHASDDFR  VFDLTNIWQVGSGDDVGK  VSSGVYSAAGYK  TTTPDSLIVGEYQTSTSDPIR  YELDYTTR  GDLWAWVPGGAAK  SPEDLSYDKR  LYTVTEAEGVR  YIINSPVSSISF  DWVQEFTTAQNLK  lytniqaysqtsepieafeaaietntk  ILLGVWASGTNTIEPEIK  LTDLIIGASIGSEDLYR  VSVTGIQNK  FIADWK  AYDAIEGAVGGK  PIWVTETGWPYVGQTWDQAAATIK  EIINIDFTK  NMVGTTLQYDNK  ETDAPTVASSR  GGFSTVANPQNEFHTYTIK  WTPTQLDWIIDGAVVRT  DAAQGTIEWAGGITDFNEGPFMGYYQSIK  IQDFMGGDGTTGAK  DATEYQYGDK  VLTGTSTTEDVGTK  DVPIMAAAGTNAIR  LLDEAGIYVISDLSEPSTSINR  WDITLYKR  GSGYQDQIDFFK  GIAYQPGGSSANLDPLADPK  AIPVGYSAADVSK  LNPGTTVTFSGK  TTFPYTSSGFAGPLLSISSSSK  GLSSSVLYGSGEK  NTDAFDIGSSSNIVIDGAK  NVLFENSVIANSENGAR  TNYGTTGLVSNITYR  YGIMVDQSYGSSSK  sltntililar  SAITAAQWETIYTYQTNFGVR  TGAGVSTQGLWHYPAK  PIATFAPSSDGTFSTTSVAAIINTFGTR  IHLSTQVDDMGLETEIYHPAGK  ISDLETHATWQSNINTR  LPAGSHYFVEMGHNGNGDFIEGLPKA  NPENSFWPAITNVATNGYDGLVIIPR  YLLGLHPDPYMFHQANLR  SGDVDQITVGSVSGK  LSLLQIWVETITQEMVR  LTNWPITSLK  SVDSYIQTETPIAQK  LQGVSNPSGSLSNGAGLGEPK  ALVEGSAFAK  VTTSYGQTVK  VVGSIAALGNWAPASGVTLSAK  LPYEYPYGEVSTTK  TVDISWFGELGVTGYVPDSQR  VATSTVTVSAGK  FNLGSAPSQATTLR  SLVTEWVTETVYETVTK  AFLDVFGALTDGR  KVEWAFSD  VDGTLSLYTEGKPWQQLFVDR  SGMGQGMMQYTTGAEPAPHNAER  VVVAENPVK  GSTLLEDFIFR  FYTDEGNFDIVGNINIPVFFIQDAIR  QDLWDAIESGNAPSWELAVQLIDEDK  FLPEEFAPLQVLGEMTLNR  NPMNYFAETEQISFQPGHIVR  GYEQATYYPGAEWTEDFDK  FWADQAHVVEMVLGQPTIHDYNK  LADLALSVAFDTPSGIPDNNLIFDPVTGPK  AGSGYSSVANVNK  VDNAATTGTSGLK  AIATIPETYEISPDLDSYNYR  SKPQYETLSASQFISAR  AVQSPQTAIK  FASQNQDMALAK  ADGTVVVTEVVQGPTVVEYVLDGK  GPLVPTANQPGPEISNEPYLDFFQYLLSLK  NSELPATLSTSYGEEEQSVPR  IGDTYSDFFDEQGR  TFFGNAIDDLAMPLFSANLNK  VLDSNGSGSNSGVIAGMNFVAQDAQSR  LVDEPVFSFYLADQDGESEVVFGGVNK  ADIAHILELLEVK  VDFLALHSYGTDGPTVLNHLLDAYSR  SFNQAIAAIELYDVAWNFGPLTFENVK  GMEAEDGVGLNIASGQQVTLNIK  DVIVSGTAISSQLPYLVIIDSVIHVLGDISEIK | 57-74  76-98  99-112  119-137  161-172  40-62  63-88  104-125  27-39  67-75  58-71  147-157  43-64  66-87  88-103  153-166  193-210  211-222  249-270  273-280  324-336  345-354  359-369  370-381  42-54  65-91  92-109  122-138  139-147  160-165  228-239  240-264  39-47  55-66  76-86  147-165  166-181  212-240  241-254  255-264  273-286  76-89  105-126  131-137  240-250  52-71  195-207  58-69  70-91  96-108  180-198  242-258  265-275  285-298  27-37  99-119  166-181  190-217  256-277  283-299  300-324  473-498  541-558  559-573  574-590  591-600  36-50  123-143  230-239  534-543  544-565  144-157  268-288  346-357  437-450  37-53  244-256  258-265  86-106  107-129  184-192  75-85  163-187  294-319  333-351  352-372  243-261  278-300  282-310  582-594  110-122  586-606  378-394  373-382  325-336  41-64  302-331  332-352  450-463  252-272  207-233  216-242  155-167  279-304  143-169  273-295  70-102 | ■  ■  ■  ■  ■  ■  ■  ■  ■  ■  ■  ■  ■  ■  ■  ■  ■  ■  ■  ■  ■  ■  ■  ■  ■  ■  ■  ■  ■  ■  ■  ■  ■  ■  ■  ■  ■  ■  ■  ■  ■  ■  ■  ■  ■  ■  ■  ■  ■  ■  ■  ■  ■  ■  ■  ■  ■ | ■  ■  ■  ■  ■  ■  ■  ■  ■  ■  ■  ■  ■  ■  ■  ■  ■  ■  ■  ■  ■  ■  ■  ■  ■  ■  ■  ■  ■  ■  ■  ■  ■  ■  ■  ■  ■  ■  ■  ■  ■  ■  ■  ■  ■  ■  ■  ■  ■  ■  ■  ■  ■  ■  ■  ■  ■  ■  ■  ■  ■  ■  ■  ■  ■  ■  ■  ■  ■  ■  ■  ■  ■  ■  ■  ■  ■  ■  ■  ■  ■ |  |

All of the peptides identified in a nano-LC/MS/MS analysis of secreted proteins from vegetative wild type cells and *Δdfg5, Δdcw1* cells are listed in the middle column. The proteins from which these peptides were derived were identified using the TurboSEQUEST software to search the proteins encoded by the *N. crassa* genome at the Broad Institute and NCU#s are shown in the first two columns. The location of the peptide within the identified protein is given in the fourth column. Whether the peptides were found in the secreted proteins from wild type cell (WT) or from the *Δdfg5, Δdcw1* (mutant) secreted proteins is denoted in the last two columns. A * by the protein name denotes a putative GPI-anchored protein.
