# Supplementary material for: The Neurospora crassa dfg5 and dcw1 Genes Encode α-1,6-Mannanases That Function in the Incorporation of Glycoproteins into the Cell Wall
Source: PLoS One. 2012 Jun 11;7(6):e38872. doi: 10.1371/journal.pone.0038872 (PMC3372484; doi:10.1371/journal.pone.0038872)
Supplement: Table S4 — Alignments for DFG5 and DCW1. Alignments of DFG5 and DCW1 from N. crassa, S. cerevisiae, C. albicans, and A. fumigatus using CLUSTAL W (1.83) multiple sequence alignment. (DOC) [file pone.0038872.s005.doc]

**Table S4**

Alignments of DFG5 and DCW1 from *N. crassa*, *S. cerevisiae*, *C. albicans*, and *A. fumigatus*

using CLUSTAL W (1.83) multiple sequence alignment.

NcDFG5 MRWNVA---VCGLMGL------LAQSATAITMDIDDTQSVKDAAATIAYG

NcDCW1 MRTTSSPRGATWLTALFAAAACLLPAANAQGYAIDTTDNIRASAKTLAFD

ScDfg5p MIVNISAKMILSICFT---FLSFFKATHAMDLDTTSKTSICDATALIQGG

ScDcw1p MLVNKV---IGLLGVLF-----ATRFTNAVELDLDNYESLQNATSLIAYG

CaDfg5p MVSLQQ--LTI---SI---LLLFTASVQSLDINVDDKDSICSAAKYVVQG

CaDcw1p MKFSI-----YLIISLF------SSFSHAIWLDTNNETTIREDCNIIAKG

Af_XP746394 MHCMQ-------LLWL----L-TLSPAYSIPLDPNDPTSIKQAAHHVAAN

Af_XP749923 MKLSSRT--WTSLVTV---VLAGQGAVTALELDINDVQSIKDAAATTAYN

* : .: .

NcDFG5 MLKYYTGNNTGDTPGNLPDP-------YYWWEAGAMFGAMVDYWWVT--G

NcDCW1 LMKFYNGNQSGQIPGILPGPPSDGKGDYYWWEGGALMGTMIDYWHLT--G

ScDfg5p MLDYYEGTRYGGTVGMFQSP-------YYWWHAGEAFGGMLENWFLC--E

ScDcw1p LMDYYTGNQYGKTVGMFSDP-------YYWWEAGGAWGCMLDYWFFM--D

CaDfg5p IWNYYEGLKYGGTVGMFAPP-------NYWWNAGEAFGGLVDFYTYCQSD

CaDcw1p LLDYYEGTKYGGVIGMFSWP-------YYWWEAGGAWGSLIDYTFYF--D

Af_XP746394 MLSHYTGMKPGDNPGNLPPP-------YYWWEAGAMFNALIDYWYLT--G

Af_XP749923 MMSNYTGNQTGQIPGKLP---------DTWWEGGAMFMTLIQYWFWT--G

: . * * . * * : **..* :::

NcDFG5 DTSYVEVTTQAIVHQAGDARDFNPANQSRTSSNDDVGFWTITAMMAAEDA

NcDCW1 DTTYNDVITQGILHQVGDNRDFQPLNFTASLGNDDQGFWGMTAMLAAENK

ScDfg5p NDTYQELLYDALLAQTGSNYDYIPSNQTMVEGNDDQGIWGITVMGAVERN

ScDcw1p NDTYNDEIIAAMIHQAGDDNDYIPLNQSTTEGNDDQAFWGIAAMTAAERN

CaDfg5p NSTLEKLIYNGMYHQAGENYNYIPSNQSMTEGNDDQGVWGMAIMEAVERN

CaDcw1p NDTLVPLITDALLYQTGDDDNYIPLNQSTTEGNDDQAFWGIAVMAAAERN

Af_XP746394 DSTWNAITTQALTWQAGHTGTFMPTNQTKTEGNDDQAFWAFAAMSAAERN

Af_XP749923 DTSYNEVTTQGMLWQKGNN-DYFPSNYSNYLGNDDQVFWGLAAMTAAELN

: : .: * * : * * : .*** .* :: * *.*

NCDFG5 FPDPP-PDQPQWLALVQAVFNQMAS-RWDDLNCGGGLRWAINDFQTGKDY

McDCW1 FPNPP-ADQPQWLALAQAVWATQAAPDRHDDTCNGGLRWQIPPTNNGYDY

ScDfg5p FTDPG-DGKPGWLAMVQAVFNTMYS-RWDSEHCGGGLRWQIFTWNSGYNY

ScDcw1p FTNPP-ENEPQWLYLAQAVFNTMAL-RWDADSCGGGLRWQIFVWNSGYDY

CaDfg5p FTEPE-S--HSWLEMVQAVFNTMNA-RWDADNCGGGLRWQIFTWNSGYDY

CaDcw1p FTNPK-DPTKAWLTLAQAVFNTMQA-RWDTETCNGGLRWQIFQWNSGYDY

Af_XP746394 FPDPDPDHGPGWLAMAQAVFNTQAA-RWDEDTCGGGLRWQIFSFNNGWNY

Af_XP749923 FPEE--DGQPSWVSLAQGVFNTQVP-RWDTSTCHGGLRWQISTYQDGYRT

*.: *: :.*.*: . * ***** * : *

NcDFG5 KNSISNGIFFNLGARLARFTGNSS-YGEWASRTWDWERSINLITD-E---

NcDCW1 KNTIANAIFFNMGARLARYTRNDT-YATWATKQFQWIYDVNYIDHDS---

ScDfg5p KNTVSNACLFQIAARLGRYTGNTT-YLEVAEQVFDWLVDVGYVVLND--T

ScDcw1p KNTVSNGALFHIAARLARYTGNQT-YVDWAEKVYEWMVGVNLISNGT--Y

CaDfg5p KNSISNGCLFHLAARLARYTGNSSVYVDTAEKVWKWMEDVGFLTEEDNGD

CaDcw1p KNSVSNGALFHLAARLARYTGNDS-YVVWAERVWDWMYGVGLLTEQN--W

Af_XP746394 KNTISNGCFFHLAARLARYTGNRT-YAEWAERVWDWTVDVGFITD-D---

Af_XP749923 KNAISNGGLFQLAARLARYTNNET-YSQWAERIWDWSATTPLLKESD---

**:::*. :*::.***.*:* * : * * : :.* :

NcDFG5 YDVKDGAHFDVTTHVCRNDSGPHVWSYNIGVFLQGAAFMYNV--STGA--

NcDCW1 WKVYDGGHVE---HNCT-DINKAQFSYSAAILVQGAAFMYNY--TEGDAA

ScDfg5p ANVFDGAEID---TNCT-DITKIEWTYNHGIVLGGLAYMYNA--TNG---

ScDcw1p KYVYDGVSID---DNCT-KVTSYQWTYNQGLLLAGSAYLYNF--T-G---

CaDfg5p VRIYDGAKIT---NNCS-SVTDLRWSYTYGVFMAGCAYLYNF--T-G---

CaDcw1p WFVYDGVKIA---NNCS-NITKYQWSYNQGLMLAGCAYLYNY--T-E---

Af_XP746394 WLFYDGADVL---LNCS-DLNRIEWTYNSGVYLLGAANMYNCYKTEG---

Af_XP749923 WTIADTTSPE---TGCT-DHGDLQWTYNYGTYISGAAYMYNF--TNG---

. * * . ::*. . : * * :** :

NcDFG5 EQETWKTRVDGLLGAVEAKFLTND-TKIIKEWYCESGFSDRGHPYQCNID

NcDCW1 TQDMWKTRIEKLTEGLFRDFFP---KGIAFELACEGR------QGACTPD

ScDfg5p -TGEWETSLTKILNGAKSYFFK---DSIMYESACQD-------YGTCNTD

ScDcw1p -SDLWHTRTKEFLNASQVFFHD----GIVYEAACQG-------PNSCNTD

CaDfg5p -DDVWLTRTNEIVQASLSYFFA---NKIMQETTCQP-------QNKCNND

CaDcw1p -EEKWYNYTIKLLESAQVFFKNISGSMVMYEAACQP-------SNSCNND

Af_XP746394 -DSRWEARTKHILQATDAFFAEDP-AMVMYERACEL-------VDTCQVD

Af_XP749923 -GDKWKKGLDGLLNTTFQRFFPFQNGMVMSEIACEP-------NMKCDRN

* : * : * *: * :

NcDFG5 QQTFKGYLLRWLSSTSQVAPYTYERINPWIRATAAAAVATCTGPVGAAAP

NcDCW1 MVSFKGYVHRWMAMVTQIAPFTRDTILPVLKTSAEAAAKQCTG-------

ScDfg5p QRTFKSIFSRMLGLTSVMAPFTRDTIDDLIKTSAEAAAKSCNG-------

ScDcw1p QRSFKAYFARFLGVTAQLVPETRNQIMSWLNTSAIAAAKSCSG-------

CaDfg5p QRSFRCLFSRCLGLTTQLAPETKDRIREVLEASAEGAAKSCSG-------

CaDcw1p QRSFKAYFSRFLGLTSVLVPQTEPVITKWLVDSANGAAGSCSG-------

Af_XP746394 QRAFKGFLARWMAAATQVAPFTYDWVMPRLRASAAAAARTCTG-------

Af_XP749923 QDCFKGFLSSWLTFMTTIVPYTSSEVVPRIQQSALAAAKQCSG-------

*: . : : :.* * : : :* .*. *.*

NcDFG5 QVDSGGIQPGFKGIDGTACGFKWTQ------TFDGWAGVGAQMNALSAVM

NcDCW1 ------------GATGRVCGFYWSGGVFVDPAVDKTTGAGEAMDVLAAVS

ScDfg5p ------------GTDGHTCGLNWQKQ-----TNDGYYGLGEQMSALEVIQ

ScDcw1p ------------GTDGHTCGLNWFN-----GTWDGMYGLGEQMSALEVMV

CaDfg5p ------------GSDGVTCGENWAID-----KWDGVYGLGEQTSALEVMM

CaDcw1p ------------GSDGVTCGLSWTDWS---QGWDGKWGLGEQMSALEVMQ

Af_XP746394 ------------GPDGAACGLKWTTG-----VWDGSEDVGLQMSALEVIQ

Af_XP749923 ------------GQNRTLCGRRWHQD-----TFDGTSSLEEQMSALSVFS

* ** * * . ..* ..

NcDFG5 YTLTHKGVGKAAKGPVTTAQGGTSKGDPGAGVTDPASRGGLAALKPITMA

NcDCW1 SLLIDE-----ADPPVTNTTGGTSKGDPNAGTGSRHA---TEPAKPITTA

ScDfg5p NLLIHD-----RPAPYKEDNGGTSKGDANAGMNSSTTNV-LQNNLNIKKG

ScDcw1p NTRALD-----KPAPYTAENGGSSVGDGAAGTQAQPT---NLAPLNITKG

CaDfg5p ALIV--------EPPLSVKTGGTNRTDYSAGTNSEDN-A-NKNELTITGK

CaDcw1p NLMVHK-----RPAPYTADTGGSSIGNPAAGYGKLTS---DATPLSIDGG

Af_XP746394 NLLVDR-----VDPPVTDATGGTSVGDPSGGMEQPDPRPP-VLTMTITGA

Af_XP749923 SSMIAHR--MQAQAPLTVDTGGTSKSNASAGTGSEQA---APQPQPVTTG

* . **:. : .* :

NcDFG5 DRVGAGIVTAILAISIVGGSVFLT----I

NcDCW1 DKAGAAMCTILLIAGG--IAIWIFMNLGD

ScDfg5p DRAGAAIITAVILSVLTGGAVWML----F

ScDcw1p SKAGAGIITAVIGISIVACALWLV----F

CaDfg5p DKAGAGVLTAIVLAVILGGAIWMI----F

CaDcw1p DKAGAGIITAIIGASLVGSCVWLI----L

Af_XP746394 DRAGAGLLTAMLGVLMIGTTGWLLY---E

Af_XP749923 DRAGAGIVTVVFLSGWIAAVTWMVY--GR

.:.**.: * :. ::
